# A scoping review on the feasibility and efficacy of mobile health applications in the management of breakthrough pain in cancer patients

**DOI:** 10.1186/s12913-026-14480-8

**Published:** 2026-04-03

**Authors:** Xinyue Zhang, Xiaoming Gao, Mengyao Gao, Siqi Hao

**Affiliations:** 1 Department of Nursing, The Second Hospital of Dalian Medical University, Dalian City, Liaoning Province 116023 China; 2 Department of Digestive Tract Oncology, The Second Hospital of Dalian Medical University, Dalian City, Liaoning Province 116023 China

**Keywords:** Breakthrough cancer pain, mHealth, Application, Scoping review, Nursing

## Abstract

**Background:**

Breakthrough cancer pain (BTcP) is a significant pain subtype affecting 60%–90% of advanced cancer patients. It is characterized by sudden onset, high intensity, and unpredictability, severely impairing function and quality of life. Mobile health (mHealth) applications are emerging as tools for real-time pain monitoring and management, but evidence of their feasibility and effectiveness remains limited.

**Objective:**

This scoping review summarizes the application status of mHealth apps in BTcP management, focusing on clinical functions, feasibility, and effectiveness.

**Methods:**

This scoping review was conducted in 2024 using the 5-step framework of Arksey and O’Malley. On October 20, 2025, a comprehensive literature search was conducted across PubMed, Embase, Web of Science, Ovid MEDLIN, CNKI, and other databases using key terms related to BTcP, mHealth, and pain management. Data were extracted and narratively synthesized.

**Results:**

A total of 10 studies from 4 countries (Spain, China, the United States, and Germany) were included. The study designs comprised 5 randomized controlled trials (RCTs), 1 quantitative study, 1 feasibility clinical trial, 2 mixed-methods studies, and 1 prospective multicenter study. The functions of the application were categorized into four domains: pain monitoring and data collection, data and management module, clinical decision-making module, and communication and alerting functions. Feasibility outcomes were evaluated in 7 studies, and effectiveness outcomes were included in 5 studies.

**Conclusion:**

Mobile health applications have demonstrated potential in back pain management; however, their feasibility and effectiveness metrics require further refinement. By analyzing included studies to develop an intervention framework for managing cancer breakthrough pain using mobile health applications, it is possible to enhance pain monitoring efficacy and the capacity for timely intervention, ultimately improving patients’ quality of life.

**Clinical trial number:**

Not applicable.

**Supplementary Information:**

The online version contains supplementary material available at 10.1186/s12913-026-14480-8.

## Background

Breakthrough cancer pain (BTcP) represents a significant subtype of cancer-related pain, characterized by sudden, severe pain episodes occurring against a backdrop of otherwise stable background pain management. These episodes can occur several times per day. Studies indicate that approximately 60.0% to 90.0% of patients with advanced cancer experience BTcP to varying degrees, with about 30.0% of these patients failing to achieve effective pain control, significantly compromising their quality of life [1–3]. This type of pain is marked by its sudden onset, high intensity, and unpredictable nature. When an episode occurs, it can severely restrict a patient’s mobility and daily functioning, and is recognized as a potential risk factor for suicide in this vulnerable population [4]. Furthermore, patients suffering from BTcP incur annual pain-related hospitalization costs that are five times higher compared to those without BTcP. This highlights that BTcP is not only a common clinical challenge but also one that is notoriously [5] difficult to diagnose and manage effectively [6].

In the clinical management of cancer pain, the diagnosis and treatment of breakthrough cancer pain (BTcP) have always been an important challenge faced jointly by patients and healthcare personnel [2]. As the primary executors of pain management, oncology healthcare professionals play a core role in pain assessment, treatment decision-making, and long-term follow-up [7]. Although there are relevant guidelines for pain assessment and management currently available [8], the management outcomes of breakthrough cancer pain remain unsatisfactory. Patients, especially during hospital discharge or home care periods, often fail to receive adequate and continuous therapeutic support [9, 10]. Studies show that more than half of cancer pain patients do not achieve sufficient pain relief, a phenomenon that is particularly prominent among discharged patients and populations in remote areas with limited access to medical resources [11]. Therefore, relying solely on intermittent in-hospital assessments and management by healthcare professionals has obvious limitations, and it is urgently necessary to utilize auxiliary tools and methods that can extend to the outpatient setting to support more systematic, continuous, and timely management of breakthrough cancer pain.

Smartphones, with their dynamic and personalized service capabilities, have become an ideal platform for health status monitoring and management. Studies [12–15] show that they not only promote collaboration between healthcare providers and patients and enhance the efficiency of personalized care but also demonstrate significant value in chronic disease management. They effectively improve patient compliance with medical follow-ups and medication adherence, improve pain control outcomes, and increase patient and healthcare provider satisfaction [16–20]. For patients with cancer-related breakthrough pain, mobile health applications can effectively overcome the spatiotemporal limitations of traditional management methods, alleviating management challenges caused by difficulties in accessing medical care, time conflicts, uneven distribution of medical resources, and economic constraints. This approach is particularly suitable for the continuous care needs of outpatients or home-based patients. Its accessibility anytime and anywhere provides an ideal response to the sudden and unpredictable clinical characteristics of breakthrough tumor pain (BTcP), offering a new avenue for real-time assessment, early intervention, and continuous monitoring of breakthrough pain [5, 21–24].

However, mobile health applications typically rely on network connectivity for functional implementation, thereby also causing issues regarding patient application accessibility, data privacy, and security. Currently, there are relatively few applications specifically developed for patients with cancer breakthrough pain (BTcP), and the vast majority of related downloadable applications available on the market have not undergone systematic feasibility and effectiveness evaluations [25, 26]. Based on this research gap, this study, guided by the framework of Arksey and O’Malley’s scoping review methodology [27], aims to systematically synthesize the feasibility and effectiveness of using mobile health applications for managing breakthrough cancer pain, to provide a scientific basis for the optimization of future mobile health applications and clinical practice.

## Method

This scoping review was conducted following the five-steps framework outlined by Arksey & O’Malley [27].

### Identifying the research question

Based on an initial review of relevant literature, the following questions were identified:

1. What are the intervention methods and functions of mobile health applications in managing cancer breakthrough pain?
2. What are the feasibility aspects and related clinical indicators for patients using mobile health applications to manage cancer breakthrough pain?
3. What are the effectiveness aspects and related clinical indicators for patients using mobile health applications to manage cancer breakthrough pain?

### Identifying relevant studies

Based on the identified research questions, the Wanfang Database, China National Knowledge Infrastructure (CNKI), VIP, Chinese Biomedical Literature Database, PubMed, Embase, Web of Science, and Ovid MEDLINE Database were retrieved. The retrieval time limit was from the database establishment to November 25, 2025. The following keywords were used: (Appendix 1: Search Strategy).

### Study selection

#### Eligibility criteria

①The study subjects were diagnosed as patients with BTcP [28]; ②The intervention involved providing mobile health technology-based interventions to BTcP patients, including interventions based on smartphones, computers, tablets, wearable devices, etc., and the article must include descriptions of specific intervention content and methods; ③Original studies such as observational studies, quasi-experimental studies, and randomized controlled trials were included, encompassing unpublished literature and gray literature.

Exclusion Criteria: ①Exclusion of cancer patients with other types of pain, as this review specifically targets the BTcP population.②Omission of non-peer-reviewed literature types, including dissertations, conference abstracts, and research letters. ③Exclusion of publications in languages other than English or Chinese.④Removal of studies for which the full text was inaccessible.

The researchers used a systematic literature search strategy to enhance the methodological quality of the scoping review [29]. They identified eight electronic databases (the Wanfang Database, CNKI, VIP Database, Chinese Biomedical Literature Database, PubMed, Embase, Web of Science, and Ovid MEDLINE). A research assistant compiled relevant articles, which a medical librarian also validated. Passive involvement studies were excluded from this study. Two reviewers (XYZ and MYG) independently screened studies according to the eligibility criteria. Ambiguous articles were read in full by two reviewers. In cases of divergent opinions on article inclusion, consensus was reached by discussions among the research team members. Expert opinions, editorials, and articles that omitted authors’ names or abstracts were excluded.

### Charting the data

Two researchers independently reviewed each article. Three data extraction tables were created in Excel, containing bibliographic details of each study. These tables collected the following information: including authors, publication year, country, study type, study population, application name, application functions, intervention methods, intervention duration, usage frequency, assessment content, and assessment indicators.

### Collating, summarizing, and reporting the results

Based on the Arksey and O’Malley framework [27], we organized the data into themes according to common characteristics (e.g., corresponding functions of mobile health applications for managing cancer breakthrough pain, feasibility and outcome measures, effectiveness and outcome measures) following qualitative content and thematic analysis. First, we carefully reviewed the research findings and extracted them. Next, we categorized similar viewpoints or research results into the most relevant themes. We presented the research findings using charts, tables, and text to summarize the extracted data. Charts were used to display the article selection process and the number of eligible articles, while tables were used to show the characteristics of included articles. The intervention methods, functions, feasibility, and outcome measures, effectiveness and outcome measures of digital health technologies were synthesized and presented in text form.

## Results

### Characteristics of included studies

**Fig. 1:**
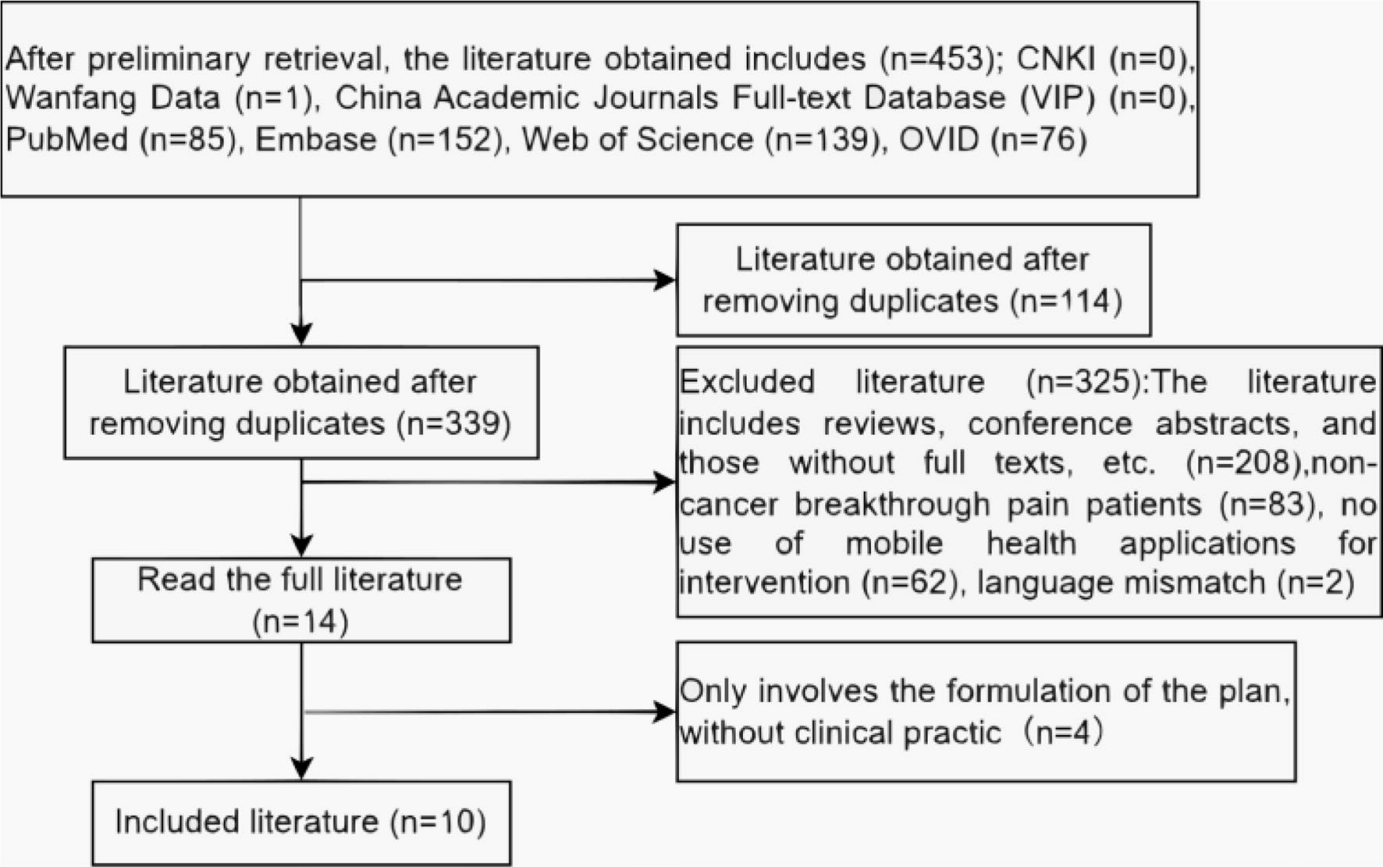
Study selection procedure

We found 453 initial articles through the electronic database search. After applying the eligibility and exclusion criteria, we retained 10 articles to comprise the body of this review (Fig. 1). The publication years of these 10 articles range from 2019 to 2025. Among them, 2 are from Spain [30, 31], 3 from China [32–34], 4 from the United States [35–38], and 1 from Germany [39]. In terms of research design, they include 5 randomized controlled trials (RCTs), 1 quantitative study, 1 feasibility clinical trial, 2 mixed-methods studies, and 1 prospective multicenter study. The basic characteristics of the included articles are presented in Table 1.

**Table 1:** Characteristics of selected studies

Research	Nation	Research Type	Person
Boceta et al. [30] 2019	Spain	Quantitative Research	175multidisciplinary physicians
Villegas et al. [31] 2021	Spain	Feasibility Clinical Trial	21 cancer patients with breakthrough pain
Yang et al. [33] 2019	China	RCT	58 outpatients with cancer pain who were discharged
Zhao et al. [32] 2024	China	RCT	42 cancer pain patients
Benze et al. [39] 2019	Germany	Prospective multicenter study	40 patients with advanced cancer
Kamdar et al. [35] 2024	United States	RCT	112 patients with advanced cancer pain
Hunter et al. [36] 2020	United States	RCT	48 children with cancer pain
Azizoddin et al. [38] 2024	United States	Mixed Methods Research	15 patients with advanced cancer pain
Sun et al. [34] 2017	China	RCT	46 cancer pain patients
LeBaron [37] et al 2022	United States	Mixed Methods Research	5 cancer patients + 5 family caregivers

### Intervention elements of mobile medical technology in the management of BTC patients

Table 2 shows the extensive differences among studies, including intervention methods, functions, intervention cycles, and usage frequency. This study focuses on cancer patients with explosive pain episodes, drawing on 10 literature sources, of which 5 are randomized controlled trials (RCTs) [32–36], with sample sizes ranging from 42 to 112 cases. Through analysis of the key design features of existing mobile health applications, it was found that they exhibit diverse adaptation strategies across application carriers. Currently, smartphones remain the mainstream platform, and most applications are compatible with Android or iOS systems [30, 31, 33, 35]; some studies have effectively improved technological accessibility by providing patients with loaned devices [34, 36, 38, 39]. To meet the needs of data collection in remote areas, integrated solutions combining wearable devices, environmental sensors, and base stations have also emerged to support multi-dimensional physiological and environmental monitoring in home settings [37]. In addition, with the development of ecological platform services, lightweight application forms such as WeChat Mini Programs have gradually been applied due to their advantages of no need for separate installation, convenient operation, and high service reach efficiency [32].

In terms of usage frequency and intervention duration, the design exhibits differentiation: high-frequency daily use (e.g., 1–2 times per day) is primarily employed for real-time symptom monitoring, such as dynamic pain assessment; medium-to-low frequency weekly use focuses on phased evaluation of indicators like quality of life. Intervention duration is predominantly short-term (2–8 weeks), aligning with the needs of acute cancer pain management, although applications supporting long-term monitoring over several months are relatively scarce. Regarding outcome measures, existing studies are mainly categorized into two types: seven studies focus on feasibility [30, 31, 34, 36–39], paying attention to technical and procedural feasibility, user satisfaction, task completion rate, and clinical recommendation willingness; five studies evaluate clinical effectiveness [32–36], with indicators covering pain scores, medication adherence, incidence and resolution rates of breakthrough pain, occurrence of adverse events, and hospitalization duration related to pain.

**Table 2:** Functions, intervention cycles, and usage frequency of mobile health applications

Research	The name of the mHealth App	The functions of the mHealth App	Intervention methods	Intervention Cycle	Usage frequency
[30]	INES.DIO	Assist in the diagnosis and monitoring of BTCP	/	/	
[31]	Pain Monitor	Instantaneous Assessment of the Pain Ecosystem	/	30 days	Record twice daily
[33]	Pain Guard	Provide continuous treatment information and feedback	Experimental group: Pain Guard application nursing; Control group: Traditional drug nursing	2 months	Record once daily
[32]	“Yao Nin You Wo”	Individualized Pharmaceutical Nursing	Experimental group: ‘Yao Nin You Wo’ WeChat mini-program; Control group: Routine nursing	4 weeks	Record at least once daily
[39]	MeQoL	Monitor patient-reported outcomes to identify pain	/	Median 99.5 days	Symptom documentation (once daily); Quality of life (once weekly)
[35]	ePAL	Active Pain Monitoring; Artificial Intelligence Algorithms for Patient Symptom Classification; Patient Education to Address Barriers to Pain Management	Experimental group: ePAL group; Control group: conventional treatment group	8weeks	Three times a week
[36]	Pain Buddy	Reduce average pain and reports of moderate to severe pain	Experimental group: Pain management using Pain Buddy; Control group: Routine care	60 days	Twice daily
[38]	STAMP + CBT	Pain Cognitive Behavioral Therapy, Remote Symptom Monitoring, and Pharmacological Support	/	2 weeks or 4weeks	Record once daily
[34]	IPMS	Pain Cognitive Behavioral Therapy, Remote Symptom Monitoring, and Pharmacological Support	Experimental group: Data collection using IPMS; Control group: Data collection via traditional methods	14 days	At least once daily
[37]	BESI-C	Support for the monitoring and management of cancer pain in the home environment	/	2weeks	On-demand usage

### Clinical functions of mobile health applications

1. Pain Monitoring and Data Collection: Among the included literature, a total of 5 studies demonstrated the functions of pain monitoring and data collection [31, 33, 34, 37, 39]. The basic approach primarily involves integrating commonly used numerical rating scales or visual analog scales in clinical practice into the application interface, enabling patients to conveniently and frequently report pain intensity dynamically. A more comprehensive assessment method embeds Ecological Momentary Assessment (EMA) into the application system, conducting real-time evaluations of the duration of pain episodes, daily or weekly frequency of occurrence, potential precipitating factors, etc. Study indicates that [31] this method sends brief assessment prompts to patients at random or fixed times throughout the day, aiming to capture real-time pain status in authentic living environments. It not only reduces recall bias commonly present in traditional retrospective records but also more accurately reveals the dynamic patterns of pain changes and their associations with contextual factors such as emotions and activities. In addition, by combining EMA with environmental sensors and wearable devices [37], it is possible to continuously collect objective behavioral data that may reflect the pain condition, providing important background information and objective corroboration for subjective assessments based on self-reports.
2. Education and Management Module: A total of 3 studies reflect this function [32, 36, 38]. It aims to directly address the root barriers caused by a lack of knowledge and insufficient self-management skills by enhancing patients’ self-efficacy. Primarily through multimedia formats such as text-illustrations and short videos, it systematically provides evidence-based information on cancer pain mechanisms, standardized use of opioid analgesics, and management of common side effects, thereby filling patients’ cognitive gaps. Relevant study [32] digitally adapted Cognitive Behavioral Therapy (CBT), converting complex psychological interventions into daily exercises that can be completed on mobile devices, thus alleviating patients’ negative psychological emotions and improving their quality of life. In addition, this module provides support for medication adherence through functions such as setting medication reminders and allowing patients to record medication usage to form electronic logs, effectively promoting the standardized implementation of treatment regimens and enhancing their ability to actively manage pain [38].
3. Clinical Decision Support Module: A total of two studies demonstrated the clinical decision support function [30, 35]. The core function of this module is to assist healthcare professionals in improving the standardization level and work efficiency of the diagnosis and treatment process through digital tools. It operates at two main levels: At the diagnostic and triage support level, its core is to convert established clinical guidelines into executable algorithm workflows. For example, Boceta et al. [30] directly embedded the Davis algorithm for diagnosing cancer-related breakthrough pain in their study, guiding physicians to complete standardized diagnostic assessments through a series of structured questions. Kamdar et al. [35] further introduced artificial intelligence rules to automatically analyze real-time symptom data reported by patients and perform risk stratification, achieving preliminary automated triage and thus optimizing clinical workflows. At the data integration and decision support level, this module aims to address the problem of information fragmentation. By automatically integrating discrete data such as patients’ daily reported pain scores, attack records, and medication logs, it generates intuitive trend charts or comprehensive dashboards. This visual data aggregation enables physicians during outpatient follow-ups to quickly and accurately grasp the overall trend of patients’ pain control and treatment response, effectively replacing the traditional communication method that relies on patients’ post-hoc recall. It provides a more objective and continuous data basis for formulating or adjusting treatment plans.
4. Communication and Early Warning Function: Four studies have demonstrated this functionality [31, 33–35]. It effectively connects patient-reported data with clinical treatment actions at the healthcare end, thereby forming a complete, data-driven management closed loop. The core of this module is an automated early warning system: when patient-submitted data exceeds pre-set clinical thresholds, the program automatically generates structured early warning reports and instantaneously sends them to designated medical and nursing teams. For instance, this function was practically applied and validated in the studies by Kamdar [35] and Villegas et al. [31], with the latter reporting that up to 86% of clinical warnings received timely follow-up from medical staff. Therefore, in subsequent clinical practice, the system can bind early warnings with a pre-defined graded response mechanism to achieve more efficient resource allocation. For low-risk warnings, the application automatically pushes relevant health education materials to patients; for medium-risk warnings, the pharmacy team is notified to conduct telephone follow-ups and medication guidance within an agreed timeframe; for high-risk warnings, healthcare personnel are directly prompted to intervene immediately for urgent assessment or treatment arrangement. This graded handling model optimizes the allocation of medical resources, enabling patients to avoid unnecessary outpatient visits or telephone disturbances, enhancing the efficiency and organization of medical communication, and providing convenience to patients.

### Feasibility and evaluation indicators of mHealth apps

Table 3 shows that a total of 7 studies [30, 31, 34, 36–39] evaluated the feasibility of mHealth Apps in pain management for patients with cancer breakthrough pain, including compliance, clinical recommendation degree, satisfaction, and technical feasibility. The results indicate that most applications performed well on key feasibility indicators, such as a high user record completion rate [39], excellent system usability scores [31], and a high willingness of healthcare providers to recommend [30]. Some studies also demonstrated the value of scenario-based innovation, such as achieving home environment monitoring through multi-device integration [40], integrating psychological and medication support modules [38], or adopting gamification design to adapt to pediatric patients [36]. Overall, these tools are generally designed to be close to clinical practical needs, focusing on core links such as symptom monitoring, medication reminders, and doctor-patient communication; most pay attention to iterative optimization based on user feedback, and have preliminarily verified basic capabilities such as offline use, cross-device compatibility, and data security at the technical level, laying an operational foundation for subsequent implementation.

However, existing evidence still has several significant limitations that restrict the generalizability of its conclusions and clinical translation prospects. First, the sample size of the studies is generally small, and the population structure is relatively homogeneous, failing to adequately include vulnerable groups such as the elderly, those with low digital literacy, or those with multiple comorbidities, resulting in representational bias due to the digital divide. Second, in terms of research methodology, only a few studies adopted a randomized controlled design, while most were single-arm or cross-sectional studies, leading to a limited evidence hierarchy and making it difficult to distinguish between the usability of the tools themselves and participants’ attention effects. Additionally, existing evaluations have primarily focused on process indicators of usage, without sufficiently linking them to clinical efficacy or long-term sustainability of use; that is, they address whether the tools are usable but have not yet sufficiently validated their effectiveness and sustained usability. Finally, some tools face limitations in technical compatibility, such as support for only a single operating system or language, hardware battery life issues, or risks of local data storage, which are practical deployment obstacles.

In conclusion, the current feasibility study has confirmed the initial acceptability and technical feasibility of mHealth applications in cancer pain management, providing a reference framework for further validation of their effectiveness. However, the existing conclusions are still primarily based on users and environments with relatively ideal conditions. Future research needs to break through the current paradigm by expanding sample sizes, extending follow-up periods, and actively incorporating diverse populations. On this basis, feasibility assessment should be more closely integrated with efficacy verification, health equity, and real-world applicability, thereby promoting the transformation of related tools from ‘usable’ to ‘effective, accessible, and sustainable’ clinical practice.

### The effectiveness and evaluation indicators of mHealth apps

A total of five studies [32–36] evaluated the efficacy of mHealth Apps, using pain relief rate, attack frequency, pain-related hospitalization time, medication adherence, drug adverse reactions, quality of life, and pain management knowledge as evaluation indicators (See Table 3). All studies reported significant improvement in core pain indicators. Among them, two studies had relatively strong evidence [33, 35]: ePAL not only reduced pain scores but also significantly decreased pain-related hospitalization rates by nearly 70%, demonstrating potential value in reducing medical burden; Pain Guard, employing a double-blind design, showed significant benefits in multiple dimensions including pain relief rate, breakthrough pain frequency, medication adherence, and quality of life. Hunter et al.‘s [22] study was the only one focusing on the pediatric population; although it did not show an advantage in daily average pain scores, it could effectively reduce the number of moderate-to-severe pain attacks. Sun [34] and Zhao et al.‘s [32] studies respectively demonstrated unique features in improving patients’ functional status, pain management knowledge, and providing pharmacist-led services. These studies collectively form the foundation of early efficacy evidence.

These studies constitute an important foundation for early efficacy evidence in this field, Its advantages are mainly reflected in three aspects: firstly, all adopted a randomized controlled trial design, providing methodological support for causal inference; secondly, the intervention functions precisely targeted core shortcomings in home-based pain management such as low adherence and poor communication; finally, the evaluation system has expanded from single pain intensity to comprehensive dimensions including medication adherence, adverse reactions, quality of life, and medical resource utilization, resulting in a more comprehensive indicator system.

However, the current evidence still has limitations. All studies were conducted at a single center with limited sample sizes (42–112 cases), and participants were mostly screened for the digital divide, which restricts the extrapolation of conclusions to broader, more heterogeneous real-world patient populations. Follow-up periods were generally short, with the longest being only 8 weeks [35] or 60 days [36], lacking medium-to-long-term (≥ 6 months) efficacy and safety data, thus unable to verify the sustainability of intervention effects. Methodologically, only one study adopted a double-blind design [33]; some studies reported insufficiently on key safety indicators [34], the independent contribution of specific functional modules [33], or details of usage compliance [32]; additionally, outcome data primarily relied on patient-initiated entry, which may introduce recall bias.

In summary, existing evidence from randomized controlled trials indicates that mobile health applications can improve cancer pain control in the short term and demonstrate potential in enhancing adherence, quality of life, and conserving medical resources. However, these conclusions are limited by small sample sizes, short follow-up periods, and high homogeneity of study populations, and thus remain preliminary evidence. Future research needs to conduct pragmatic randomized controlled trials with multi-center, large-sample, long-term designs, actively including vulnerable populations with lower digital literacy, in order to generate high-level evidence with strong confirmatory power and fair generalizability, thereby providing a solid basis for the systematic application of such tools in clinical practice.

**Table 3:** Feasibility and Efficacy of Mobile Health Applications and Related Clinical Indicators

Research	Evaluation Content	Evaluation Metrics
[30]	Feasibility	Usability, acceptability, and tool utility
[31]	Feasibility	Compliance; Usability
[33]	Feasibility; effectiveness	Feasibility: Satisfaction Effectiveness: Pain relief rate; Incidence of BTcP; Improvement in quality of life; Incidence of adverse reactions
[32]	effectiveness	Pain scoring; medication adherence; incidence and remission rates of BTcP; incidence of adverse events
[39]	Feasibility	Adherence; Usability
[35]	Feasibility	Pain-related scoring, pain-related length of hospital stay
[36]	Effectiveness; Feasibility	Effectiveness: Frequency of moderate-to-severe pain episodes; Feasibility: Participant compliance
[38]	Feasibility	Completion rate, acceptability
[34]	Feasibility; Effectiveness	Feasibility: Compliance; Acceptability; Effectiveness: Pain intensity, functional status, pain-related management knowledge
[37]	Feasibility	Technical and procedural feasibility; results of semi-structured interviews

## Discussion

Through this review, we aim to address three questions: (1) What are the intervention methods and functions of mobile health applications in the management of cancer breakthrough pain? (2) What are the feasibility and related clinical indicators of using mobile health applications to manage cancer breakthrough pain patients? (3) What are the effectiveness and related clinical indicators of using mobile health applications to manage cancer breakthrough pain patients? In this scoping review, we reviewed the categories and functions of mobile health applications (mHealth Apps), including three approaches: single-application interventions [30, 31, 33–36, 38, 39], WeChat mini-programs [32], and environmental sensors + Bluetooth positioning + wearable devices [40]. Compared with traditional pain management methods, mobile health technology, through multiple carriers such as smartphones, tablets, and internet platforms, achieves real-time management and monitoring of breakthrough pain, significantly improving patients’ quality of life. It not only meets the needs of cancer breakthrough pain patients for instant pain assessment but also provides them with more convenient, efficient, and personalized experiences through innovative digital technologies, indicating that using mobile health applications to manage cancer breakthrough pain is feasible and effective.

Research has found that the current number of relevant randomized controlled trials is limited, and most are single-center, small-sample studies with a generally short intervention period (2–14 weeks) and sample sizes mostly ranging from 42 to 112 cases. Regarding intervention compliance, there are differences in results across studies: some shorter-cycle studies reported high patient usage compliance and favorable outcomes, whereas in longer-intervention studies, a trend of gradually decreasing compliance over time was observed. In terms of usage frequency, most studies set evaluation frequencies (such as 1–2 times per day) that were well accepted, but a few feedback indicated that overly frequent reminders or push notifications might increase patient burden. In summary, the current evidence base remains weak, and there is an urgent need to further standardize cancer breakthrough pain management protocols based on mobile health technology, scientifically set intervention cycles, and follow-up nodes, thereby more accurately evaluating pain relief and enhancing the sustainability and effectiveness of intervention measures.

In terms of sample size, application cycle, and frequency of intervention, the included studies found that there is currently a limited number of relevant randomized controlled trials, most of which are single-center and small-sample studies. The intervention cycles are generally short, with frequencies categorized as high-frequency (twice daily) and medium-to-low frequency (once weekly). The results of the included literature show variations: some shorter-cycle studies demonstrated high patient adherence and good efficacy, whereas in longer-intervention cycle studies, a trend of gradually decreasing adherence over time was observed [39]. Regarding usage frequency, the assessment frequencies set by most studies (e.g., 1–2 times daily) are widely accepted; however, a few feedback reports indicate that overly frequent reminders or push notifications may increase patient burden [38]. In summary, the current evidence base remains weak, and there is an urgent need to further standardize cancer breakthrough pain management protocols based on mobile health technology. Scientifically setting intervention cycles and follow-up nodes is necessary to more accurately evaluate pain relief and improve the sustainability and effectiveness of intervention measures.

The applied functions of the included literature in the review are primarily categorized into four modules: monitoring and evaluation [31, 33, 34, 37, 39], education and management [32, 36, 38], clinical decision support [30, 35], and communication and alerting [31, 33–35]. However, high-quality monitoring does not automatically translate into effective management. Most studies have only verified the feasibility of data collection but have failed to sufficiently demonstrate that these data are systematically and effectively utilized to guide clinical decisions and ultimately improve patient outcomes. Regarding the education and management module, while functions such as Cognitive Behavioral Therapy (CBT) and medication reminders are commonly described, their presentation is often static and unidirectional information push. Few studies evaluate whether users truly understand, internalize, and apply this knowledge. The effectiveness of this module is frequently reported in conjunction with the overall application’s performance, lacking isolated validation of the active contribution of the educational components themselves. The clinical decision support module, although clearly describing the functions of algorithms (e.g., Davies algorithm) and visualization dashboards, rarely measures the integration of these tools into real clinical workflows and healthcare provider adoption rates. Critical factors influencing their actual utility—such as whether the tools add extra workload, whether clinicians trust and act upon algorithm recommendations—are almost absent from existing functional descriptions. In the communication and alerting module, the tiered alert system is a core innovation for closed-loop management; however, its major limitation lies in the fact that the success of this function entirely depends on a pre-established, efficient internal human response system within hospitals. For example, Villegas’ study reported an 86% response rate to alerts, demonstrating conceptual feasibility but without evaluating sustainability and scalability. Under the normal state of resource constraints, this highly human-dependent module may be the most vulnerable link in the entire system. In summary, the existing literature on application functions exhibits a significant characteristic of emphasizing technical implementation over integration validation. Studies have amply demonstrated what can be done, but severely lack in-depth exploration of whether and why they remain effective in real complex scenarios. Future functional research must shift from descriptive to explanatory, focusing on evaluating the integration of each functional module into clinical workflows, adoption rates among healthcare providers and patients, and cost-effectiveness. Particular attention needs to be paid to revealing their adaptability and effectiveness for vulnerable populations such as the elderly.

In the analysis of feasibility and effectiveness results, the diagnostic criteria for feasibility primarily include user compliance rate [31, 34, 38–40], system usability [30, 31], and satisfaction scores [33, 34], healthcare provider acceptance [30], and deployment success rate [26, 30, 36, 40]; the diagnostic criteria for effectiveness mainly include pain relief [32, 34–36], shortened hospital stay [35], improved medication adherence [32, 33] and reduced medication adverse reactions [32, 33], and enhanced patient quality of life [34]. In the included overall studies, the feasibility and effectiveness of the application were validated, which, to a certain extent has formulated research protocols for the subsequent clinical application of mobile applications in managing cancer breakthrough pain. However, there are also limitations in the included literature. Among the 10 included studies, only 5 were strictly randomized controlled trials [32–36], while the rest were mostly pilot or feasibility studies. Although such preliminary studies are necessary, they were not designed to verify effectiveness themselves, and their positive results may be biased due to small sample sizes and lack of blinding. Limitations in study scale and duration: Most studies had limited sample sizes and relatively short intervention periods (mostly 4–12 weeks). This restricts the precise estimation of effect size and also prevents the evaluation of the long-term effects, tolerance, and potential side effects of the application. Insufficient representativeness of population and scenarios: Studies were mostly conducted in tertiary medical centers with good digital literacy and medical resources, with insufficient attention to vulnerable groups such as the elderly, low-income individuals, or those facing difficulties in using digital technologies, limiting the extrapolation of conclusions. Based on the above assessment, future research should focus on: (1) Conducting larger-sample, longer-duration multicenter randomized controlled trials (RCTs) to confirm efficacy and evaluate cost-effectiveness; (2) Paying attention to differential effects in different patient subgroups; (3) Deeply exploring which intervention combinations are most effective for specific outcomes; (4) Establishing a standardized core set of feasibility indicators to facilitate cross-study comparisons. In summary, mobile health applications for managing cancer breakthrough pain are a promising but still requiring mature validation field. Current evidence is sufficient to support its continued development and more rigorous evaluation, but it is still some distance from widespread clinical routine application.

In feasibility studies of mobile health applications, network connectivity methods are primarily categorized into three types: systems with continuous or high-frequency connectivity [31, 33–35, 37] can enable real-time alerts and consultations, offering the highest responsiveness; however, they may exacerbate the digital divide and affect reliability due to unstable networks or patient burden. Systems with intermittent or offline recording [30, 36, 38, 39] reduce network dependency, enhancing availability and inclusivity in resource-limited settings, with the core value lying in supporting data collection and patient self-monitoring. Provider-mediated or platform-integrated communication [32] leverages mature ecosystems to improve accessibility but faces external risks such as data privacy issues, platform dependency, and information delays. The selection of these connectivity methods essentially represents a critical trade-off between technical feasibility and clinical accessibility, immediate intervention and patient burden, development convenience and long-term controllability, directly impacting the study’s inclusivity, data quality, and clinical translation potential. Choosing connectivity methods is, in fact, a value prioritization made by researchers between immediacy and inclusivity, technical idealism and practical constraints, development convenience and academic sovereignty. A study proven ‘feasible’ only in highly connected environments has fragile conclusions; whereas completely avoiding real-time connectivity may limit the clinical potential of the application. Future research should abandon adherence to single models, shift toward adaptive design, and honestly report actual performance data and ethical compromises under different connectivity strategies. Only in this way can mobile health research advance toward truly robust and equitable clinical translation.

In mHealth applications targeting cancer patients, protecting patient data privacy is the foundation for ensuring clinical credibility and ethical compliance. Currently, researchers have strengthened privacy protection through various strategies and achieved positive feedback: Azizoddin et al. [38] implemented strict informed consent and HIPAA-compliant storage in their pain management application, resulting in high patient satisfaction scores (87% of items rated ≥ 4/5); Hunter et al. [36] used de-identified unique IDs to record children’s pain data, effectively avoiding direct exposure of personal information; Kamdar et al. [35] enhanced patient recognition of application security by transmitting pain information through end-to-end encryption; LeBaron et al.‘s [37] study showed that a multi-device data stream collection method processed through de-identification led most participants to not feel significant privacy threats (mean score 1.9/5). Despite this, existing applications still have certain limitations in privacy protection. Some patients expressed concerns about continuous environmental monitoring, feeling monitored by the government. This reflects that passive, uninterrupted data collection methods may still raise user concerns about privacy breaches. Zhao et al. [32], while improving accessibility through a WeChat Mini Program, face external risks such as data privacy, platform dependency, and information delays. Additionally, Benze et al. [39] encountered issues during device recovery, including data irrecoverability due to device loss, transmission interruptions caused by unstable networks, and insufficient support for privacy settings for elderly or cognitively impaired groups, further exposing the limitations of current protection mechanisms. Therefore, in clinical practice and subsequent development, privacy protection strategies need to be further optimized: technically, cloud encryption combined with local backup should be adopted to enhance the stability and security of data transmission; procedurally, device management standards and data lifecycle supervision should be established; simultaneously, privacy notification and control mechanisms that are understandable and operable for different groups should be designed, thereby advancing pain management effectiveness while genuinely safeguarding patient data privacy and trust.

Unlike previous research conclusions, the included literature generally reflects that mobile health applications have high feasibility and are easy to use, which is consistent with the research conclusions of Villegas et al. [31] and multiple studies [41–43]. These studies all confirm that regardless of cancer patients’ age, educational background, or previous technology usage experience, they can smoothly use the relevant applications. However, through a systematic analysis of the age groups in the included literature, existing mHealth research has blind spots and systematic exclusion regarding the inclusiveness of the elderly and populations with low information literacy. Except for pediatric studies, the average age of participants is mostly between 50 and 65 years, with no specific inclusion or analysis of elderly patients (≥ 75 years), those with cognitive limitations, or those unfamiliar with technology. All studies implicitly impose a high digital literacy threshold, requiring proficient use of smartphones, excluding individuals with cognitive/visual impairments, and relying on specific language interfaces. The reported high feasibility conclusions may only apply to relatively young, healthy, and technologically adaptable subgroups of patients, resulting in significant selection bias. This exposes potential usage inequities in existing mobile health research: by inadvertently screening the most benefited groups through technical access criteria while excluding the most vulnerable, digitally less capable elderly patients. This not only limits the external validity of research conclusions but may also exacerbate rather than alleviate existing health inequalities through technology. Therefore, the current evidence cannot prove that these applications are truly feasible for a broad and heterogeneous population of cancer pain patients, especially the elderly and those with low information literacy. To bridge this digital divide, future research needs to adopt a comprehensive approach incorporating multiple factors to enhance patients’ electronic health literacy and capabilities. This requires moving beyond mere technological delivery and adopting the digital support system proposed by Kong et al. [44], which includes key components such as training and social support. In this process, digital inclusiveness should be a core ethical and scientific indicator, involving proactive design of age-friendly interfaces [45], provision of hybrid support models combining telephone and paper-based assistance, and stratified reporting of data across different capability subgroups, thereby truly promoting fair and effective digital health solutions.

### Suggestions for an integrated framework for BTcP management based on mHealth

In terms of monitoring core clinical features, ecological momentary assessment (EMA) [31, 33] should be integrated to systematically capture multidimensional data, including pain intensity, location, frequency, duration, as well as emotional and fatigue-related metrics, thereby minimizing recall bias. Monitoring frequency should combine routine daily assessments with pain event-driven triggers, and push strategies should be optimized to reduce burden [38, 39]. Patient-provider interaction pathways should establish a tiered closed-loop response: through the implementation of alert mechanisms [33, 36], moderate-to-severe pain triggers prompt patients with medication guidance and follow-up reminders; for complex cases, alerts are securely escalated to healthcare teams to facilitate clinical follow-up [31, 35]. Regarding data governance, privacy concerns, and multi-stakeholder data sharing requirements raised in Lebaron et al.‘s research [37] must be addressed, with clear ownership definitions, encrypted transmission protocols, and data visualization tailored to provide customized views for different stakeholders. Finally, feasibility metrics should extend beyond short-term satisfaction to encompass long-term technical reliability, changes in user compliance rates, actual impact on clinical outcomes as revealed by multiple studies, and scalability and fairness across diverse healthcare settings and cultural contexts, with particular attention to remote rural areas. This framework aims to systematically integrate effective components from existing evidence, address identified gaps, and provide a clear blueprint for developing the next generation of robust, effective, and clinically integrable BTcP management tools.

## Conclusion

Existing research has preliminarily confirmed the feasibility and effectiveness of mobile health applications in the management of breakthrough cancer pain. However, due to the limited number of included randomized controlled trials, which are mostly small-sample, single-center, and short-term follow-up studies, the evidence strength and extrapolability of their conclusions require further validation. Based on a systematic review of the strengths and limitations of the included studies, this review proposes a corresponding research optimization framework. Future studies should conduct large-sample, multi-center, long-term clinical trials to further confirm their intervention effects, thereby effectively reducing the clinical management burden on healthcare providers and improving the overall quality of life of patients with breakthrough cancer pain.

## Supplementary Information

Below is the link to the electronic supplementary material.

Supplementary Material 1

## Data Availability

Data is provided within the manuscript.
