# Supplementary material for: A scoping review on the feasibility and efficacy of mobile health applications in the management of breakthrough pain in cancer patients
Source: BMC Health Serv Res. 2026 Apr 3;26:690. doi: 10.1186/s12913-026-14480-8 (PMC13169952; doi:10.1186/s12913-026-14480-8)
Supplement: Supplementary file 1 — Supplementary Material 1 [file 12913_2026_14480_MOESM1_ESM.docx]

Appendix 1: Search Strategy

PubMed

1. ("Mobile Applications"[Mesh]OR "Mobile Health"[Title/Abstract] OR "mHealth"[Title/Abstract] OR "Smartphone"[Title/Abstract] OR   "Cell Phone"[Title/Abstract] OR"App*"[Title/Abstract] OR "Digital Health"[Title/Abstract])
2. ("Breakthrough Pain"[Mesh] OR "Breakthrough Cancer Pain"[Title/Abstract] OR "BTcP"[Title/Abstract] OR "Cancer pain"[Mesh] )
3. ("Neoplasms"[Mesh] OR "Cancer"[Title/Abstract] OR "Oncolog*"[Title/Abstract] OR "Tumor"[Title/Abstract] OR "Malignan*"[Title/Abstract])
4. #1 AND #2 AND #3 ("Mobile Applications"[Mesh] OR "Mobile Health"[Title/Abstract] OR "mHealth"[Title/Abstract] OR "Smartphone"[Title/Abstract] OR "Cell Phone"[Title/Abstract] OR "App"[Title/Abstract] OR "Apps"[Title/Abstract] OR "Digital Health"[Title/Abstract] ) AND ( "Breakthrough Cancer Pain"[Title/Abstract] OR ("Breakthrough Pain"[Mesh] OR "Cancer Pain"[Mesh] ORAND ("Neoplasms"[Mesh] OR cancer[Title/Abstract] OR oncolog*[Title/Abstract])) OR "BTcP"[Title/Abstract] )

Wanfang Database
主题:(移动应用 OR 移动健康 OR 移动医疗 OR 智能手机 OR APP OR 应用程序 OR 数字健康) AND 主题:(爆发痛 OR 癌性爆发痛 OR 爆发性癌痛 OR 癌性疼痛 OR BTcP) AND 主题:(癌症 OR 肿瘤 OR 恶性肿瘤 OR 癌)

CNKI
(SU='移动应用' OR SU='移动健康' OR SU='移动医疗' OR SU='智能手机' OR SU='APP' OR SU='应用程序' OR SU='数字健康') AND (SU='爆发痛' OR SU='癌性爆发痛' OR SU='爆发性癌痛' OR SU='癌性疼痛' OR SU='BTcP') AND (SU='癌症' OR SU='肿瘤' OR SU='恶性肿瘤' OR SU='癌')

VIP
(U=移动应用 OR U=移动健康 OR U=移动医疗 OR U=智能手机 OR U=APP OR U=应用程序 OR U=数字健康) AND (U=爆发痛 OR U=癌性爆发痛 OR U=爆发性癌痛 OR U=癌性疼痛 OR U=BTcP) AND (U=癌症 OR U=肿瘤 OR U=恶性肿瘤 OR U=癌)

Chinese Biomedical Literature Database
(移动应用 OR 移动健康 OR 移动医疗 OR 智能手机 OR APP OR 应用程序 OR 数字健康) AND (爆发痛 OR 癌性爆发痛 OR 爆发性癌痛 OR 癌性疼痛 OR BTcP) AND (癌症 OR 肿瘤 OR 恶性肿瘤 OR 癌) [默认在全部字段检索]

Embase
('mobile application'/exp OR 'mobile health'/de OR 'mHealth':ti,ab OR 'smartphone'/exp OR 'cell phone'/exp OR 'app':ti,ab OR 'digital health':ti,ab) AND ('breakthrough pain'/exp OR 'breakthrough cancer pain':ti,ab OR 'BTcP':ti,ab OR 'cancer pain'/exp) AND ('neoplasm'/exp OR 'cancer':ti,ab OR 'oncolog':ti,ab OR 'tumor':ti,ab OR 'malignan*':ti,ab)

Web of Science
TS=("mobile applications" OR "mobile health" OR "mHealth" OR "smartphone" OR "cell phone" OR "app" OR "digital health") AND TS=("breakthrough pain" OR "breakthrough cancer pain" OR "BTcP" OR "cancer pain") AND TS=("neoplasms" OR "cancer" OR "oncolog" OR "tumor" OR "malignan*")

Ovid MEDLINE
(exp "Mobile Applications"/ OR "Mobile Health".ab,ti,kf. OR "mHealth".ab,ti,kf. OR exp "Smartphone"/ OR exp "Cell Phone"/ OR "app".ab,ti,kf. OR "Digital Health".ab,ti,kf.) AND (exp "Breakthrough Pain"/ OR "Breakthrough Cancer Pain".ab,ti,kf. OR "BTcP".ab,ti,kf. OR exp "Cancer Pain"/) AND (exp "Neoplasms"/ OR "cancer".ab,ti,kf. OR "oncolog".ab,ti,kf. OR "tumor".ab,ti,kf. OR "malignan*".ab,ti,kf.)
